# Editorial: Invasive and non-invasive vagal nerve stimulation for the brain

**DOI:** 10.3389/fneur.2023.1269851

**Published:** 2023-08-30

**Authors:** Yutian Yu, Yu Wang

**Affiliations:** ^1^Acupuncture Department, Beijing Shijitan Hospital, Capital Medical University, Beijing, China; ^2^Ninth School of Clinical Medicine, Peking University, Beijing, China; ^3^Institute of Acupuncture and Moxibustion, China Academy of Chinese Medical Sciences, Beijing, China

**Keywords:** vagal nerve stimulation (VNS), non-invasive, brain diseases, autonomic consequences, working memory (WM), headache, insomnia

The origins of vagal nerve stimulation (VNS) may be dated back to the 1880s. Over the course of the 20th century, research pertaining to VNS has been closely associated with its inherent invasiveness. Based on auricular acupuncture (AA) and conventional VNS, non-invasive VNS entered neuromodulation in the late 2000s. In brain diseases, it is well-known that the major confirmed indicators of VNS are refractory epilepsy and treatment-resistant depression. Due to the neuroanatomic features of the vagal nerve and autonomic consequences, de facto VNS can be applied to more diseases, including multiple neurologic and psychiatric disorders. Numerous methods, performed on both animals and humans, have been employed in VNS studies of the brain, expanding its indicators and elucidating inspirational and novel insights into this unique therapeutic strategy.

The objective of this Research Topic was to gather new research on both invasive and non-invasive forms of VNS for brain illnesses and autonomic effects, beyond the established indicators of epilepsy and depression. Five articles, including four Original Research articles and one Review article, were accepted.

Gurtubay et al. focused on the autonomic consequences of transcutaneous auricular vagus nerve stimulation (taVNS) by recording the P300 cognitive event-related potential (ERP). The results of their study indicate that there are varying levels of facilitation observed within a certain timeframe after taVNS. Previous studies investigating the impact of taVNS on the P300 ERP have shown diverse findings. Drawing a definitive conclusion on this relationship is challenging, given the lack of a universally defined system for taVNS and the significant variability observed in the stimulation modalities used, including variations in targets and settings. The authors optimized a variety of stimulation settings, including a specially designed earbud stimulator, an expanded stimulating surface, simultaneous stimulation over the cymba and cavum conchae, a delayed biphasic pulse burst, and current-controlled stimulation, which altered the output voltage and ensured that a predetermined electrical dose was administered. Modulation of the P300 ERP in healthy individuals may be achieved by targeting vagal nerve fibers via taVNS under specified stimulation parameters. Insufficient research has been conducted in the literature regarding the best settings for the modulatory function of taVNS on P300 ERP and the dependency between these settings. However, the available data does provide some parameters that may be easily optimized to enhance the reliability of results in future studies.

The study conducted by Tian et al. investigated the synergistic impact of taVNS and 0.1 Hz slow-paced breathing (SPB) on working memory (WM). taVNS has been shown to have the potential to improve WM performance. Previous studies have shown evidence that SPB, which refers to breathing at a rate of approximately six breaths per minute, with a frequency of 0.1 Hz, may have significant effects on both physical wellbeing and cognitive abilities by modifying the autonomic afferent activity. The present research examined the potential synergistic effects of taVNS and SPB on WM performance. The findings presented in the study demonstrated that both taVNS and taVNS combined with SPB resulted in enhanced WM performance. However, there was no statistically significant difference seen between the SPB group and the sham group.

Simmonds et al. conducted an open-label observational trial and a meta-analysis to examine the efficacy of non-invasive vagus nerve stimulation (nVNS) in individuals with medically resistant chronic cluster headache (CCH). Several studies have shown that nVNS may not be efficacious in the treatment of CH, despite assertions to the contrary. Hence, the objective of their study was to assess the safety and effectiveness of nVNS in individuals diagnosed with CCH. The research provides evidence of the potential advantages of nVNS in CCH, since it significantly decreases both the frequency and severity of headaches. In order to provide a more comprehensive understanding of the impact, the authors have suggested that validation of the positive outcomes associated with VNS, as shown in some open-label studies but not universally, necessitates the implementation of randomized sham-controlled trials.

In recent times, there has been growing recognition of the potential of neuromodulation methods as a non-pharmacological approach to treating migraine. Song et al. conducted a comprehensive analysis of randomized controlled trials pertaining to nVNS for the treatment of migraine; the primary objective of their research was to evaluate the effectiveness, safety, and tolerance of nVNS in this context. The findings of the meta-analysis indicate that the use of non-invasive cervical vagus nerve stimulation (n-cVNS) had a substantial effect on the 50% responder rate. However, it did not result in a reduction in migraine or headache days. On the other hand, the use of low-frequency non-invasive auricular vagus nerve stimulation (n-aVNS) resulted in a significant decrease in the frequency of migraine days and the severity of headaches. However, it did not have a significant impact on the number of days acute medication was taken each month. In addition, it was shown that n-cVNS had a high level of safety and tolerability among the majority of patients. The findings suggest that nVNS has potential as an effective therapeutic approach for the management of migraines.

There is a lack of assurance about whether taVNS effectively alleviates insomnia via the modulation of functional connections associated with the thalamus. Thus, the study conducted by Zhao et al. aimed to examine the modulatory effects of taVNS on the resting state functional connectivity (RSFC) of the thalamus. Before the administration of taVNS therapy, the RSFC of the thalamus, right insula, and inferior frontal gyrus exhibited higher values in patients diagnosed with insomnia disorder (ID) compared to those without the disease, who served as healthy controls. The RSFC of patients exhibited a notable reduction in the connections between the thalamus and the right angular gyrus, the left anterior cingulate gyrus, and the precuneus after the taVNS intervention. This research elucidates the immediate impact of taVNS on the functional connections between the thalamus and other regions of the brain in individuals with ID.

In summary, the articles collected in this Research Topic included non-invasive VNS studies on autonomic consequences, working memory, headache, and insomnia. We are happy to see that researchers are employing invasive and non-invasive VNS for brain indicators other than epilepsy and depression. Due to the anatomical properties of the vagal nerve and the principal mechanism of VNS (1), more brain indicators of VNS may emerge in the future, particularly in neurodevelopmental disorders and neurodegenerative diseases. Moreover, closed-loop invasive or non-invasive VNS has become a new direction in the development of VNS (2). It is anticipated that further research endeavors will shed light on these subjects.

## Author contributions

YY: Conceptualization, Supervision, Validation, Writing—original draft, Writing—review and editing. YW: Writing—original draft.
